# Use of a 1.0 Tesla open scanner for cardiovascular magnetic resonance evaluation of pediatric and congenital heart disease

**DOI:** 10.1186/1532-429X-17-S1-Q99

**Published:** 2015-02-03

**Authors:** Jimmy C Lu, James C Nielsen, Layne Morowitz, Muzammil Musani, Maryam Ghadimi Mahani, Prachi P Agarwal, Adam L Dorfman

**Affiliations:** 1 Pediatrics and Communicable Diseases, University of Michigan, Ann Arbor, MI, USA; 2 Stony Brook University, Stony Brook, NY, USA; 3 Radiology, University of Michigan, Ann Arbor, MI, USA

## Background

Open cardiovascular magnetic resonance (CMR) scanners offer the potential for imaging patients with claustrophobia or large body size, but at a lower 1.0 Tesla magnetic field. There is a paucity of data in the pediatric and congenital heart disease population. This study aimed to evaluate the efficacy of open CMR for evaluation of pediatric and congenital heart disease.

## Methods

This retrospective, cross-sectional study included all patients ≤18 years old or with congenital heart disease who underwent CMR on a Panorama High Field Open scanner (Philips, Best, The Netherlands) at two centers from 2012-2014. Indications for CMR, clinical questions and demographic data were extracted from the medical record and requisitions. A single experienced observer graded image quality (4-excellent, 3-adequate, 2-poor, 1-nondiagnostic), and ability to answer the clinical question (4-answer with confidence, 3-answer adequately, 2-low certainty, 1-nondiagnostic).

## Results

A total of 64 patients (median 17.4 years old, 61% male) were included, with 5 patients under 10 years of age. Congenital heart disease was present in 32 (50%), with tetralogy of Fallot and bicuspid aortic valve the most common diagnoses. Patients were scanned on open CMR due to scheduling/equipment issues in 51 (80%), claustrophobia in 7 (11%), and patient size in 3 (5%). In patients with claustrophobia, 4/7 had failed an attempt on a different scanner, but completed the study without sedation on open CMR. All patients had adequate or excellent image quality on black blood, phase contrast, magnetic resonance angiography, and late gadolinium enhancement imaging. There was poor image quality in 3/63 (5%) patients with cine images, and 4/15 (27%) patients with coronary artery imaging. The clinical question was answered adequately in all but 2 patients (Table 1); 1 patient with a Fontan had coil artifact limiting evaluation of RV volume, and in 1 patient the right coronary artery origin was not well seen.

**Table 1:** Ability to answer clinical questions with open CMR (N=64). Data given as number (percent).

Clinical question	1 (nondiagnostic)	2 (low certainty)	3 (adequately)	4 (with confidence)
Ventricular size/function	0	1 (2%)	10 (16%)	50 (82%)

Pulmonary arteries	0	0	1 (7%)	13 (93%)

Regurgitant fraction	0	0	5 (23%)	17 (77%)

LGE	0	0	4 (25%)	12 (75%)

Aortic root dimensions	0	0	3 (20%)	12 (80%)

Coronary arteries	0	1 (8%)	4 (33%)	7 (58%)

Aortic arch anatomy	0	0	1 (10%)	9 (90%)

Qp:Qs ratio	0	0	2 (25%)	6 (75%)

Pulmonary veins	0	0	0	4 (100%)

LGE, late gadolinium enhancement. Qp:Qs, ratio of pulmonary to systemic blood flow.

## Conclusions

Open CMR scanners can effectively evaluate pediatric and congenital heart disease, including patients with claustrophobia and larger body size. Although minor artifacts may be present, the majority of clinical questions can be answered adequately, with some limitations with coronary artery imaging. Further evaluation is necessary to optimize protocols and image quality.

## Funding

The authors have no relevant disclosures.

